# Effectiveness of bedside staplers in bariatric robotic procedures

**DOI:** 10.1007/s00464-024-11045-w

**Published:** 2024-07-17

**Authors:** Benjamin L. Clapp, Helmuth Billy, Rami E. Lutfi, I.-Wen Pan

**Affiliations:** 1 Department of Surgery, Texas Tech Paul Foster School of Medicine, El Paso, USA; 2 Ventura Advanced Surgical Associates, Ventura, USA; 3 Chicago Medical School, Rosalind Franklin University of Medicine and Science, North Chicago, US; 4 Medtronic, Plc, Boston, USA

**Keywords:** Bedside staplers, Gastric bypass, Gastric sleeves, Robotic staplers, Surgical staplers

## Abstract

**Background:**

Few studies have evaluated the use of laparoscopic staplers in robotic procedures (bedside stapling, BS). This study aims to evaluate the effectiveness of BS compared with robotic staplers (RS) in bariatric robotic procedures.

**Methods:**

Patients who underwent robotic sleeve gastrectomy or gastric bypass elective procedures between 1/1/2021 and 12/31/2021 were extracted from PINC AI™ Healthcare Data. The following clinical outcomes were compared: blood transfusion, bleeding, anastomotic leak, intensive care unit (ICU) visit, and 30-day readmission, operating room (OR) time, inpatient costs, and length of stay. We evaluated baseline balance in BS and RS and bivariate association between covariates and outcomes using Chi-square or Fisher exact test and *t*-test or ANOVA. Multivariable general linear mixed models (GLMMs) with respective gamma or binomial distribution and log-link function were used to obtain adjusted outcomes variations between BS and RS.

**Results:**

Total of 7268 discharges were included with 1603 (22.1%) BS and 5665 (77.9%) RS cases. RS cases consisted of a higher number of patients who were Hispanic (17.0% vs. 9.4%), had Medicaid (26.9% vs. 19.4%) and underwent sleeve gastrectomy (68.4% vs. 53.5%). Higher proportions of RS cases were done by providers in Northeast region (35.5% vs. 24.3%), smaller size (< 500 beds; 71.1% vs. 52.3%), and teaching hospitals (59.4% vs. 39%). The adjusted outcomes variations demonstrated that patients that had RS were significantly more likely to have blood transfusions, ICU stays, increased ORT (19 min) and costs ($1273). Sensitivity analysis showed similar results, except no significant differences in blood transfusion rates in both groups.

**Conclusions:**

Bedside staplers significantly reduce healthcare resource utilization with equivalent effectiveness and fewer ICU stays compared to robotic staplers.

**Graphical abstract:**

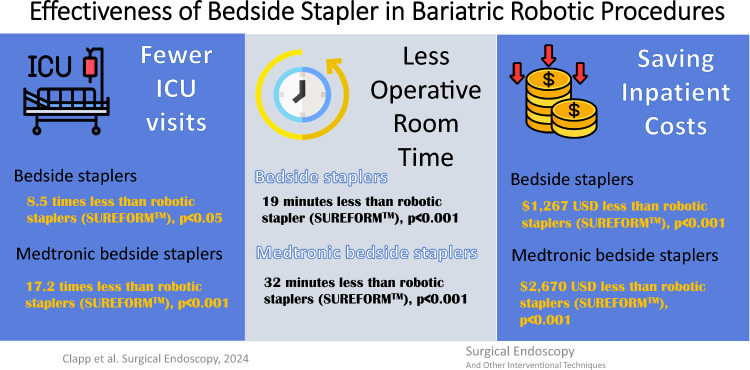

**Supplementary Information:**

The online version contains supplementary material available at 10.1007/s00464-024-11045-w.

Surgical stapling is ubiquitous in abdominal surgery. Surgical staplers have advanced through the years and have now become automated. They now include built in safety features as well as the capability to sense tissue thickness and resistance to the device. This feedback in real time can allow the surgeon to modify staple cartridge size to accommodate thicker or thinner tissue. The safety profile of these staplers is very high, with an estimated failure rate of 1 in 8000 firings [1]. Even then, most of those events are not clinically significant, and are usually just an equipment failure with no risk to the patient. We previously reported on the safety of stapling devices and found that the most common adverse event reported to the Food and Drug Administration was failure to fire for both Medtronic and Ethicon. Failure to fire can be time consuming and frustrating to the surgeon but has little chance of harming the patient. The efficacy of these staplers is proven daily, and data from the Metabolic and Bariatric Surgery Accreditation and Quality Improvement Program (MBSAQIP) demonstrates very low leak and bleeding rates [2]. The MBSAQIP does not have the granularity to determine if stapling devices are directly responsible for leaks or bleeding, but with the rise of the sleeve gastrectomy (SG), which is a purely stapling procedure, most surgeons recognize the role of the stapler in these two complications.

In the last decade, a third company has entered the arena of surgical stapling. Intuitive Surgical introduced the robotic platforms stapler in 2012 [3]. The first iteration of this robotic stapler (RS) was the Endowrist™ and later it has been refined to the Sureform™. There is little data reported on this stapler, but by evaluating the MBSAQIP, suppositions about its safety can be made. According to the latest American Society of Metabolic and Bariatric Surgeons estimates, robotic-assisted cases made up 30% of total bariatric operations in 2022 [4]. There is no statistically significant increase in leakage in these cases compared to non-robotic, again demonstrating equivalent safety. However, these observations are muddled by the fact that not all robotic surgeons use the robotic stapler. The type of stapler used is not reported in the MBSAQIP, so a robotic case may or may not have used a robotic stapler. There are a few reasons for this, including surgeon comfort level with their traditional bedside staplers and distrust in the first-generation robotic stapler.

Few studies have evaluated results of non-robotic bedside staplers (BS) used in robotic procedures [5–7]. In addition, a previous study showed that robotic staplers need more reloads to complete the gastric pouch and the overall stapling costs were higher than bedside staplers [8]. The objective of this study is to evaluate the effectiveness of bedside staplers compared with robotic staplers during bariatric robotic-assisted procedures using a nationwide hospital-based database.

## Materials and methods

### Data sources

Data were extracted from the PINC AI™ Healthcare Data (PHD). The PHD comprises U.S. hospital-based, service-level, all-payor information on inpatient discharge [9]. More than 1400 hospitals/healthcare systems contribute data to the PHD with more than 9 million visits per year since 2012, representing approximately 25% of annual United States inpatient admissions. The PHD contains information on hospital and visit characteristics, admitting and attending physician specialties, healthcare payers, and patient data, including demographics, disease states, diagnoses, costs, medications, and device details from standard hospital discharge billing files. The PHD is de-identified in accordance with the HIPAA Privacy Rule. This study was determined exempt from full board review by Sterling IRB.

### Study population

**Fig. 1:**
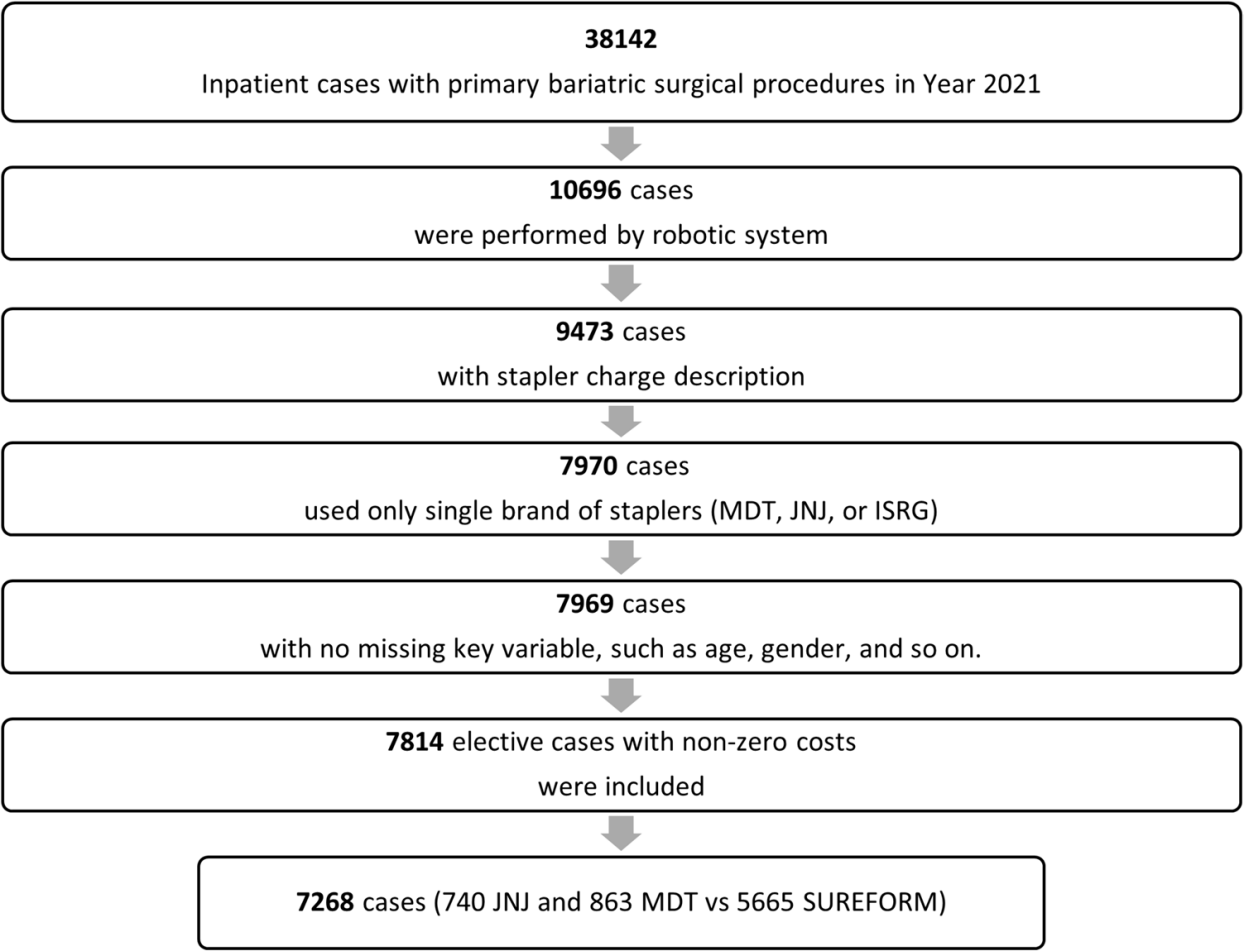
Study cohort selection flow chart

Patients who underwent primary sleeve gastrectomy (SG) or Roux-en-Y gastric bypass (RYGB) with a robotic system in the inpatient setting between 1/1/2021 and 12/31/2021 were obtained from PINC AI™ Healthcare Data. Inclusion criteria were patients whose procedure used BS and RS, had all the key variables, and had non-zero costs (Fig. 1). Robotic procedures were defined as an inpatient claim with a secondary procedure code (International Classification of Diseases version-10 [ICD 10 PCS]) or any claim with a Current Procedure Terminology (CPT) code indicated as a robotic procedure or patients charged with robotic supplies. These included CPT code S2900, ICD-10 code 8E0W4CZ.

### Study design

**Fig. 2:**
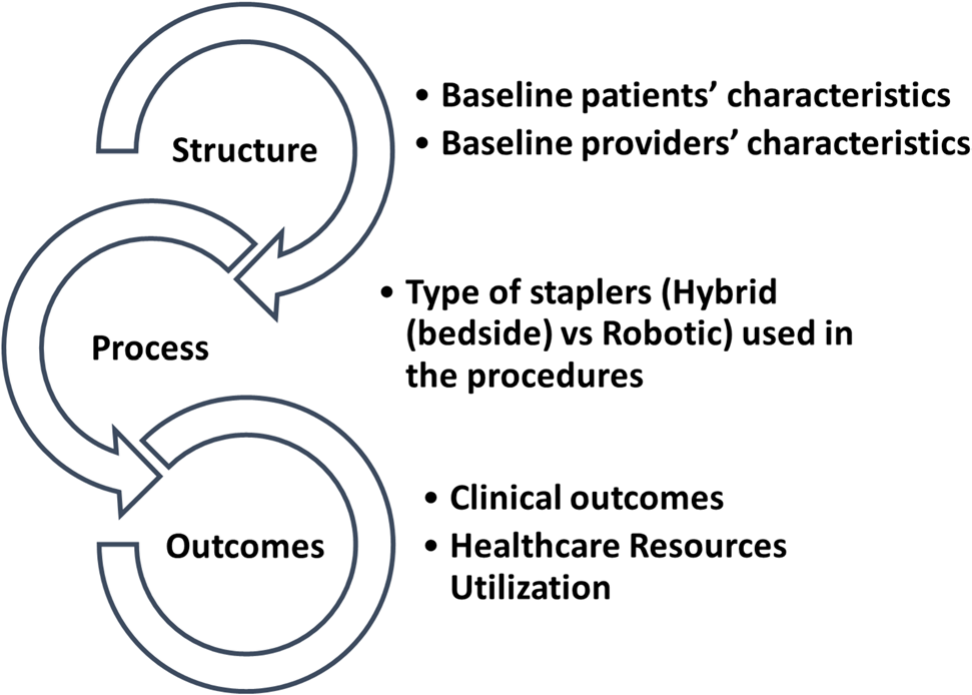
Donabedian’s structure-process-outcomes quality framework [10]

This study is based on Donabedian A^4^. “Structure-Process-Outcomes Quality Framework” to evaluate outcomes (clinical outcomes and healthcare resource utilization) effectiveness of bariatric robotic procedures (Fig. 2) [10].

### Clinical outcomes and healthcare resource utilization

Clinical outcomes included the rates of blood transfusion, bleeding, anastomotic leak, intensive care unit (ICU) visits, and 30-day urgent and emergency room readmission. We used ICD-10 diagnosis and procedures codes and CPT codes to identify blood transfusion, bleeding, and anastomotic leak from patients’ inpatient claim file. Premier ‘READMIT’ file and charge master files were used to identify ICU and readmission. Healthcare resource utilization included operating room (OR) time in minutes obtained from the hospital charge file, costs (in US dollars), and length of stay (in days).

### Patients and hospital characteristics, and type of staplers used

Patient characteristics included age group (< 40, 40–54, 55–64, and 65), gender, race and ethnicity (Non-Hispanic White, Non-Hispanic Black, Hispanic, and others), payer (Medicare, Medicaid, Private, and others), obese diagnosis, sleeve or bypass procedures, Charlson comorbidity index (0, 1–2, and 3^+^), and APR severity (Minor/Median vs Major/Extreme). Provider characteristics included region (Northeast, Midwest, South, and West), urban or rural, bed size (< 500 beds vs ≥ 500 beds), teaching status, and bariatric procedure annual volume.

The key variable evaluated in the study is the type of stapler. We grouped staplers into two groups: bedside staplers included Johnson and Johnson Ethicon™ or Echelon™ staplers and Medtronic Signia™, Endo Gia™, Tri-staple™ staplers; the robotic staplers included the latest Intuitive SUREFORM™ staplers. The manual/intelligent bedside staplers (MIBS) subgroup only included Medtronic beside staplers. All types of staplers were used product name/product number as text searching through the hospital change master file.

All study codes are listed in Supplementary Table 1.

### Statistical analysis

Effectiveness was measured by clinical outcomes and healthcare resource utilization. The covariates to evaluate effectiveness included baseline patient and provider characteristics. We evaluated baseline balance in BS and RS and the bivariate association between covariates and outcomes using Chi-square or Fisher exact test and *t*-test or ANOVA. Multivariable general linear mixed models (GLMMs) with respective gamma or binomial distribution and log-link function were used to obtain the variations of adjusted outcomes and healthcare resources utilization between BS and RS. Additionally, a subgroup analysis was performed to analyze the effectiveness of manual/intelligent bedside staplers.

### Sensitivity analysis

For testing the robustness of the results, two additional modeling approaches for sensitivity analyses are used. First, we used propensity score matching methods to adjust baseline imbalance for two types of staplers. We performed a 1:1 match between bedside staplers and robotic staplers with a maximum caliper width of 0.2 for the absolute probability using the nearest neighbor technique without replacement. All baseline patients and provider characteristics were input into a logistic regression model for propensity score matching. The chi-squared, fisher exact, or paired t-test was used to examine the outcomes of the post-matched cases [11]. Secondly, we used propensity scores covariate adjustment multivariable general linear mixed models to estimate the outcome variations between types of staplers used. The propensity score calculation was based on all baseline patients and provider characteristics. The statistical significance was determined if *p*-value < 0.05. All data management and analyses were conducted using SAS 9.4 software (SAS Institute Inc, Cary, NC) using 2-sided statistical tests.

## Results

### Baseline patients and hospital characteristics

A total of 7268 discharges met the inclusion criteria and included 1603 (22.1%) BS and 5665 (77.9%) RS cases. RS cases, compared to BS, consisted of a higher number of patients who were Hispanic (17.0% vs. 9.4%), had Medicaid (26.9% vs. 19.4%), and underwent sleeve gastrectomy (68.4% vs. 53.5%). Compared to BS, higher proportions of RS cases were done by providers in Northeast region (35.5% vs. 24.3%), urban (97.2% vs. 92.4%), smaller hospital size (< 500 beds; 71.1% vs. 52.3%), and teaching hospitals (59.4% vs. 39%). MIBS, a subgroup of BS, had similar patient and hospital characteristics distribution. Compared to MIBS, a higher proportion of RS cases were under age 40 (40.9% vs 35.1%), had less than three comorbidities (93.6% vs 91.3%), and were performed in lower volume hospitals (46.5% vs 27.7%) (Table 1).

**Table 1:** Patient and provider characteristics

Description	Study cohort		BS		RS		*P*-value	MIBS		*P*-value
	No	%	No	%	No	%	BS vs RS	No	%	MIBS vs RS
Total	7268		1603		5665			863		
Age group							0.083			**0.011**
< 40	2913	40.08	599	37.4	2314	40.9		303	35.1	
40–54	2937	40.41	678	42.3	2259	39.9		370	42.9	
55–64	1061	14.60	248	15.5	813	14.4		137	15.9	
> 65	357	4.91	78	4.9	279	4.9		53	6.1	
Gender							0.469			0.649
Female	5963	82.04	1325	82.7	4638	81.9		701	81.2	
Male	1305	17.96	278	17.3	1027	18.1		162	18.8	
Race and ethnicity							**< 0.001**			**< 0.001**
Non-Hispanic white	3638	50.06	856	53.4	2782	49.1		492	57.0	
Non-Hispanic black	1420	19.54	375	23.4	1045	18.5		189	21.9	
Hispanic	1116	15.35	151	9.4	965	17.0		73	8.5	
Others/unknown	1094	15.05	221	13.8	873	15.4		109	12.6	
Payer							**< 0.001**			**< 0.001**
Medicare	897	12.34	241	15.0	656	11.6		141	16.3	
Medicaid	1834	25.23	311	19.4	1523	26.9		142	16.5	
Private/commercial	3993	54.94	911	56.8	3082	54.4		505	58.5	
Other	544	7.48	140	8.7	404	7.1		75	8.7	
Obesity diagnosis							0.380			0.786
No	371	5.10	75	4.7	296	5.2		47	5.5	
Yes	6897	94.90	1528	95.3	5369	94.8		816	94.6	
Procedure category							**< 0.001**			**< 0.001**
Gastric bypass	2538	34.92	746	46.5	1792	31.6		443	51.3	
Gastric sleeve	4730	65.08	857	53.5	3873	68.4		420	48.7	
Charlson comorbidities							0.056			**0.028**
No	3655	50.29	791	49.3	2864	50.6		411	47.6	
1 and 2	3120	42.93	682	42.6	2438	43.0		377	43.7	
3^+^	493	6.78	130	8.1	363	6.4		75	8.7	
APR severity							0.627			0.355
Minor/moderate	7173	98.69	1584	98.8	5589	98.7		848	98.3	
Major/extreme	95	1.31	19	1.2	76	1.3		15	1.7	
Provider region							** < 0.001**			** < 0.001**
Northeast	2398	32.99	389	24.3	2009	35.5		165	19.1	
Midwest	1326	18.24	317	19.8	1009	17.8		271	31.4	
South	2683	36.92	752	46.9	1931	34.1		394	45.7	
West	861	11.85	145	9.1	716	12.6		33	3.8	
Provider location							** < 0.001**			**< 0.001**
Urban	6985	96.11	1481	92.4	5504	97.2		818	94.8	
Rural	283	3.89	122	7.6	161	2.8		45	5.2	
Provider bed size							**< 0.001**			** < 0.001**
1: < 500 beds	4867	66.96	838	52.3	4029	71.1		254	29.4	
2: 500 + beds	2401	33.04	765	47.7	1636	28.9		609	70.6	
Provider teaching status							**< 0.001**			**< 0.001**
1. Yes	3990	54.90	625	39.0	3365	59.4		371	43.0	
2. No	3278	45.10	978	61.0	2300	40.6		492	57.0	
Provider bariatric procedure volume							0.385			** < 0.001**
1. High (> median 115 cases in 2021)	3869	53.23	838	52.3	3031	53.5		624	72.3	
2. Low (≤median 115 cases in 2021)	3399	46.77	765	47.7	2634	46.5		239	27.7	

The bold shows statistical significance, *p*-value < 0.05

*RS* robotic staplers, *BS* bedside staplers, *MIBS* manual/intelligent bedside stapler

### The effectiveness of clinical outcomes and healthcare resource utilization

The crude rates of blood transfusion and ICU stays were higher for RS compared to BS (1.2% vs 0.5%, *p* = 0.015; 0.64% vs 0.12%, *p* = 0.012). The proportion of ICU stays was also higher in the RS group compared to the MIBS group. (0.64% vs 0%, *p* = 0.011). For other clinical outcomes (bleeding, anastomotic leak, and readmission), bedside staplers had equivalent results as robotic staplers. The unadjusted OR time was higher when RS were used (152.3 ± 64.9 (mean ± standard deviation) minutes) compared to BS (137.9 ± 54.1 min) as well as MIBS (129.9 ± 51.3 min). Bedside staplers ($14,202 ± $5209) were less costly compared to robotic staplers ($15,156 ± $6141) (Table 2).

**Table 2:** Unadjusted clinical outcomes and health care resources utilization by types of staplers

Type of staplers	BS	RS	*p*-value	MIBS	*p*-value
Clinical outcomes	%	%	BS vs RS	%	MIBS vs RS
Blood transfusion	0.50	1.20	**0.015**	0.46	0.054
Bleeding	1.56	1.48	0.823	2.09	0.183
Anastomotic leak	0.12	0.37	0.203	0.12	0.348
ICU	0.12	0.64	**0.012**	0.00	**0.011**
Readmission	8.92	9.14	0.784	9.39	0.819

Healthcare resources utilization	Mean (STD)/median (IQR)	Mean (STD)/median (IQR)	BS vs RS	Mean (STD)/median (IQR)	MIBS vs RS
OR time	137.9 (54.1)/132 (105, 165)	152.3 (64.9)/150 (119, 180)	**< 0.001**	129.9(51.3)/120(100, 150)	**< 0.001**
Inpatient cost	$14,202 ($5209)/$13,582 ($10,569, $16,802)	$15,156 ($6141)/$14,116 ($11,775, $17,346)	**< 0.001**	$13,450($5196)/$13,183($10,230, $15,824)	**< 0.001**
Length of stay	1.64 (1.8)/1 (1.2)	1.42 (0.96)/1 (1,2)	**< 0.001**	1.71(2.33)	**< 0.001**

The bold shows statistical signficance, *p*-value < 0.05

*RS* robotic staplers, *BS* bedside staplers, *MIBS* manual/intelligent bedside staplers, *ICU* intensive care unit, *STD* standard deviation, *IQR* interquartile range, *OR* operating room

After balancing baseline patient and provider variation between RS and BS, the adjusted outcomes variations between RS and BS groups showed that the RS group was significantly more likely to have blood transfusions odds ratio [(OR) (95% confidence interval (CI)): 2.4 (1.1, 5.0), *p* = 0.02], ICU stays [OR 8.5 (95% CI 1.9, 37.0)], increased operative time [OR 19 (95% CI 17.9, 20.2)], and costs [$1273 ($1191, $1355)] US dollars with a lower length of stay [OR − 0.26 (95% CI − 0.27, − 0.24) days, *p* < 0.001] compared to the BS group. Compared to RS, MIBS patients had 31.6 min shorter operating time (95% CI 29.4, 33.8) and $2670 less inpatient costs (95% CI $2518, $2822), but slightly longer length of stay (0.16 days, 95% CI 0.15, 0.16, *p* = 0.002) (Tables 3 and 4).

**Table 3:** Adjusted clinical outcomes variations between robotic staplers and the compared bedside staplers

Clinical outcomes	Main model		Sensitivity analyses	
	Multivariable GLMMs^a^		Propensity score covariate adjustment GLMMs^b^	
	Odds ratio (95% CI)	*P*-value	Odds Ratio (95% CI)	*P*-value
RS vs. BS				
Blood transfusion	2.4 (1.1–5.0)	**0.022**	2.0 (0.9–4.2)	0.084
Bleeding	0.9 (0.6–1.5)	0.821	1.1 (0.7–1.8)	0.672
Anastomotic leak	3.6 (0.7–19.4)	0.132	2.8 (0.6–12.6)	0.180
ICU	8.5 (1.9–37.0)	**0.004**	8.2 (1.9–34.9)	**0.004**
30 days readmission	1.1 (0.9–1.3)	0.385	1.1 (0.9–1.4)	0.252
RS vs. MIBS				
Blood transfusion	2.4 (0.9–6.7)	0.088	1.5 (0.5–4.4)	0.478
Bleeding	0.8 (0.4–1.3)	0.302	0.9 (0.5–1.7)	0.849
Anastomotic Leak	6.5 (0.6–72.4)	0.129	3.0 (0.3, 26.4)	0.314
ICU	17.2 (1.1–275.3)	**0.044**	33.8 (2.0–565.4)	**0.014**
30 days readmission	1.0 (0.8–1.3)	0.993	1.1 (0.8–1.4)	0.582

The bold shows statistical signficance, *p*-value < 0.05

*GLMMs* general linear mixed model, *CI* confidence interval, *RS* robotic staplers, *BS* bedside staplers, *MIBS* manual/intelligent bedside staplers, *ICU* intensive care unit

^a^Multivariable GLMMs: Multivariable general linear model with log link and binomial family function

^b^Propensity score covariate adjustment GLMMs: propensity scores adjusted general linear model with log link and binomial family function

**Table 4:** Adjusted variations of health care resources utilization between robotic staplers and the compared bedside staplers manual/intelligent bedside staplers and robotic staplers

Resources utilization	Main model		Sensitivity analysis	
	Multivariable GLMMs^a^		Propensity score covariate adjustment GLMMs^b^	
Difference = RS–BS	Difference (95% CI)	*P*-value	Difference (95% CI)	*P*-value
OR time in Minutes	19.0 (17.9, 20.2)	**< 0.001**	16.8 (16.5, 17.1)	**< 0.001**
Cost in USD	$1273 ($1191, $1355)	**< 0.001**	$861 ($847, $875)	**< 0.001**
LOS in days	− 0.26 (− 0.27, − 0.24)	**< 0.001**	− 0.14 (− 0.14, − 0.14)	**< 0.001**
Difference = RS–MIBS				
OR time in min	31.6 (29.4, 33.8)	**< 0.001**	22.5 (22.1, 22.9)	**< 0.001**
Cost in USD	$2670 ($2518, $2822)	**< 0.001**	$1697 ($1669, $1725)	**< 0.001**
LOS in days	− 0.16 (− 0.16, − 0.15)	**0.002**	− 0.09 (− 0.25, − 0.07)	**0.003**

The bold shows statistical signficance, *p*-value < 0.05

*GLMMs* general linear mixed model, *CI* confidence interval, *RS* robotic staplers, *BS* bedside staplers, *MIBS* manual/intelligent bedside staplers, *OR* odds ratio, *LOS* length of stay, *USD* United States dollars

^a^Multivariable GLMMs: multivariable general linear mixed model with log link and Gamma family function

^b^Propensity score covariate adjustment GLMMs: using propensity scores to replace covariates in the GLMMs

### Sensitivity analysis

Our sensitivity analysis–propensity score covariate adjustment GLMMs showed similar results except no significant differences in blood transfusion rates between BS and RS (Tables 3 and 4). The propensity scores matching (PSM) method did not obtain enough matched pairs for an unbiased estimation of outcomes variations, for BS versus RS (1517 pairs, 94.6% of cases were matched) and for MIBS versus RS (657 pairs, 76.1% of cases were matched). However, the results of PSM were similar to the other two approaches (Table 5).

**Table 5:** The results of the propensity score matched cohort^a^

Type of staplers	RS vs BS (base group)^b^		RS vs MIBS (base group)^c^	
Clinical outcomes	OR (95% CI)	*P*-value	OR (95% CI)	*P*-value
Blood transfusion	2.0 (0.9–4.2)	0.084	1.5(0.5–4.4)	0.478
Bleeding	1.1 (0.7–1.8)	0.672	0.9 (0.5–1.7)	0.849
Anastomotic leak	2.8 (0.6–12.6)	0.180	3.0 (0.4–26.4)	0.314
ICU visit	8.2 (1.9–34.9)	**0.004**	33.8 (2.0–565.4)	**0.014**
Readmission	1.1 (0.9–1.4)	0.252	1.1 (0.8–1.4)	0.582

Resources utilization	OR (95% CI)	*P*-value	OR (95% CI)	*P*-value
Operating room time in minutes	16.8 (16.5, 17.1)	**< 0.001**	22.5 (22.1, 22.9)	**< 0.001**
Costs in USD	$861 ($847, $875)	**< 0.001**	$1697 ($1669, $1725)	**< 0.001**
LOS in days	− 0.14 (− 0.14, − 0.14)	**< 0.001**	− 0.09 (− 0.25, − 0.07)	**0.003**

The bold shows statistical signficance, *p*-value < 0.05

*GLMMs* general linear mixed model, *CI* confidence interval, *RS* robotic staplers, *BS* bedside staplers, *MIBS* manual/intelligent bedside staplers, *OR* odds ratio, *LOS* length of stay *USD* United States dollars, *ICU* intensive care unit

^a^Covariates for propensity score matching included procedure and all patient and provider characteristics, 657 manual/intelligent bedside stapling and robotic stapling cases were paired

^b^1517 (94.6%) of 1603 bedside and robotic stapling cases were matched

^c^657 (76.1%) of 863 manual/intelligent bedside stapling and robotic stapling cases were paired

## Discussion

The important findings of this study were that bedside staplers had better outcomes than robotic staplers in terms of lower rates of blood transfusion, less ICU stays, shorter operative time and lower costs. The difference in blood transfusion was not shown in the GLMM sensitivity analysis. It should be noted that these findings are from a single year (2021) using data from a hospital-based database. But they may be generalizable to the bariatric population as a whole, since this data is collected on a national level. This study helps to clarify outcomes related to the type of stapler in robotic-assisted cases. Up until now, it was difficult to determine if complications in robotic surgery were secondary to the type of stapler used. This is because most papers use the MBSAQIP database for short-term outcomes. But there is no way to determine what type of stapler was used in the MBSAQIP database, as that is not reported data. That is why the PINC AI™ was used to evaluate this, as each stapling company’s product could be individually evaluated. However, it is important to note that this data is from 2021, and in the last 3 years surgeon use of RS may have dramatically increased [4]. In fact, the ASMBS Task Force in bariatric volume reported a rate of 30% robotic-assisted cases in 2022.

Bedside stapling was the norm for robotic-assisted bariatric operation until Intuitive released their first generation of endoscopic staplers. But even then, many surgeons continued to use beside staplers. Intuitive has continuously improved their stapler and released a newer version. The robotic platform also can be modified with software updates to improve performance of the RS. The rate of bedside stapling will likely decrease over time in robotic-assisted cases as time goes by.

The question remains; however, will surgeons continue to use BS with the Intuitive robotic platform? Bedside staplers have a long track record and have improved continuously over the years. There is force feedback in these staplers and tissue sensing technology. Many surgeons have their assistant fire the stapler at the bedside and prefer to have a skilled assistant there. There is also a familiarity factor and mid to late career surgeons have used these staplers literally tens of thousands of times and may be resistant to changing from a product they are very comfortable with.

### Study limitation

Due to small or zero events for ICU visits in the comparison of Medtronic staplers versus SUREFORM, we used a modified model (Firth or Fisher exact) to analyze and estimate the rate of ICU visits. As a result, the 95% confidence interval was wide. Another limitation is that the hospital-based database does not include the granularity of staple load (height) and staple line reinforcement. These factors can affect the formation of staples and outcomes and we were unable to account for these two factors.

## Conclusion

Bedside staplers significantly reduce healthcare resource utilization with equivalent effectiveness and fewer ICU stays compared to robotic staplers.

## Supplementary Information

Below is the link to the electronic supplementary material.

Supplementary file1 (DOCX 15 kb)
