# Supplementary material for: Effectiveness of bedside staplers in bariatric robotic procedures
Source: Surg Endosc. 2024 Jul 17;38(9):5310–8. doi: 10.1007/s00464-024-11045-w (PMC11362250; doi:10.1007/s00464-024-11045-w)
Supplement: Supplementary file 1 — Supplementary file1 (DOCX 15 kb) [file 464_2024_11045_MOESM1_ESM.docx]

Supplemental:

Table 1: study code list

| Variable defined | Codes | Type of codes |
| --- | --- | --- |
| sleeve gastrectomy | 0DB64Z3 | ICD 10 PCS |
| gastric bypass | 0D164ZA | ICD 10 PCS |
| obese | E66.01 | ICD 10 DX |
| Blood transfusion | 30233H0, 30233N0, 30243H0, 30243N0, 30233H1, 30243H1, 30233N1, 30233P1, 30243N1, 30243P1, 30230H0, 30230H1, 30230J0, 30230J1, 30230K0, 30230K1, 30230L0, 30230L1, 30230M0, 30230M1, 30230N0, 30230N1, 30230P0, 30230P1, 30230Q0, 30230Q1, 30230R0, 30230R1, 30230S0, 30230T0, 30230V0, 30230W0, 30233J0, 30233J1, 30233K0, 30233K1, 30233L0, 30233L1, 30233M0, 30233M1, 30233P0, 30233Q0, 30233Q1, 30233R0, 30233R1, 30233S0, 30233T0, 30233V0, 30233W0, 30240H0, 30240H1, 30240J0, 30240J1, 30240K0, 30240K1, 30240L0, 30240L1, 30240M0, 30240M1, 30240N0, 30240N1, 30240P0, 30240P1, 30240Q0, 30240Q1, 30240R0, 30240R1, 30240S0, 30240T0, 30240V0, 30240W0, 30243J0, 30243J1, 30243K0, 30243K1, 30243L0, 30243L1, 30243M0, 30243M1, 30243P0, 30243Q0, 30243Q1, 30243R0, 30243R1, 30243S0, 30243T0, 30243V0, 30243W0, 30250H0, 30250H1, 30250J0, 30250J1, 30250K0, 30250K1, 30250L0, 30250L1, 30250M0, 30250M1, 30250N0, 30250N1, 30250P0, 30250P1, 30250Q0, 30250Q1, 30250R0, 30250R1, 30250S0, 30250T0, 30250V0, 30250W0, 30253H0, 30253H1, 30253J0, 30253J1, 30253K0, 30253K1, 30253L0, 30253L1, 30253M0, 30253M1, 30253N0, 30253N1, 30253P0, 30253P1, 30253Q0, 30253Q1, 30253R0, 30253R1, 30253S0, 30253T0, 30253V0, 30253W0, 30260H0, 30260H1, 30260J0, 30260J1, 30260K0, 30260K1, 30260L0, 30260L1, 30260M0, 30260M1, 30260N0, 30260N1, 30260P0, 30260P1, 30260Q0, 30260Q1, 30260R0, 30260R1, 30260S0, 30260T0, 30260V0, 30260W0, 30263H0, 30263H1, 30263J0, 30263J1, 30263K0, 30263K1, 30263L0, 30263L1, 30263M0, 30263M1, 30263N0, 30263N1, 30263P0, 30263P1, 30263Q0, 30263Q1, 30263R0, 30263R1, 30263S0, 30263T0, 30263V0, 30263W0, 3E030GC, 3E033GC, 3E040GC, 3E043GC, 3E050GC, 3E053GC, 3E060GC, 3E063GC, | ICD 10 PCS |
|  | 36430, 86890, 86891, 86985, P9010, P9011, P9012, P9016, P9017, P9019, P9020, P9021, P9022, P9023, P9032, P9033, P9034, P9035, P9036, P9037, P9038, P9039, P9040, P9043, P9044, P9048, P9050, P9051, P9052, P9053, P9054, P9055, P9056, P9057, P9058, P9059, P9060, P9070, P9071, P9072, | CPT |
| Bleeding | D62 , R58, K92.2, K92.1, K91.840, K91.841, K91.870, K91.871, N99.62, N99.820, N99.821, | ICD 10 DX |
| Anastomotic leak | K63.2, K65.1, K91.3, K91.89, K91.81, K91.83 | ICD 10 DX |
